# CXCL12 in astrocytes contributes to bone cancer pain through CXCR4-mediated neuronal sensitization and glial activation in rat spinal cord

**DOI:** 10.1186/1742-2094-11-75

**Published:** 2014-04-16

**Authors:** Wen Shen, Xue-Ming Hu, Yan-Nan Liu, Yuan Han, Li-Ping Chen, Chen-Chen Wang, Chao Song

**Affiliations:** 1 Department of Pain Medicine, The Affiliated Hospital of Xuzhou Medical College, 99 Huaihai West Road, Xuzhou 221002, People’s Republic of China; 2 Jiangsu Province Key Laboratory of Anesthesiology, Xuzhou Medical College, 209 Tongshan Road, Xuzhou 221002, People’s Republic of China

**Keywords:** CXCL12, CXCR4, Chemokine, Neurons, Astrocytes, Microglia, Spinal cord, Bone cancer pain

## Abstract

**Background:**

Previous studies have demonstrated that chemokine CXCL12 and its receptor CXCR4 are critical for pain sensitization, but the mechanisms involved are not clear. In this study, we investigated the specific cellular mechanisms of CXCL12/CXCR4 chemokine signaling in the development and maintenance of bone cancer pain after tumor cell implantation (TCI).

**Methods:**

TCI in the tibial cavity of rats was used to establish a bone cancer pain model. Mechanical allodynia and thermal hyperalgesia were determined by measuring the paw withdrawal threshold and latency, respectively. The protein expression and cellular localization of CXCL12 and CXCR4 were detected by western blot and immunofluorescence staining. The sensitization of neurons, activation of astrocytes and microglia were examined by observing the immunofluorescence intensity of c-Fos, GFAP and IBA1.

**Results:**

Our results demonstrated that CXCL12 was upregulated in a time-related manner, both in the dorsal root ganglia and spinal cord after TCI. Spinal CXCL12 was predominately expressed in astrocytes, and an intrathecal injection of astrocyte metabolic inhibitor fluorocitrate or selective JNK inhibitor SP600125 abolished TCI-induced CXCL12 production. A single intrathecal injection of a CXCL12 neutralizing antibody (10 μg/10 μl) at day 10 after TCI transiently reversed bone cancer pain in a dose-dependent manner. Whereas repetitive intrathecal administration of a CXCL12 neutralizing antibody (10 μg/10 μl, once a day from day 3 to 5 after TCI) significantly delayed the onset of TCI-induced pain behaviors for nearly five days. Spinal CXCR4 was also upregulated after TCI and colocalized with neurons, astrocytes and microglia. Blocking CXCR4 suppressed TCI-induced activation of neurons, astrocytes and microglia in the spinal cord at day 14. Repeated intrathecal administration of AMD3100 (5 μg/10 μl, once a day for three days) significantly delayed and suppressed the initiation and persistence of bone cancer pain in the early phase (at day 5, 6 and 7 after TCI) and in the late phase (at day 12, 13 and 14 after TCI) of bone cancer, respectively.

**Conclusions:**

Taken together, these results demonstrate that CXCL12/CXCR4 signaling contributed to the development and maintenance of bone cancer pain via sensitizing neurons and activating astrocytes and microglia. Additionally, this chemokine signaling may be a potential target for treating bone cancer pain.

## Introduction

Bone cancer pain (BCP) is a common symptom among patients with advanced breast, lung, and prostate cancer as these tumors have a prominent affinity to metastasize to the skeleton. In epidemiological studies, 75 to 90% of patients with metastatic or advanced stage cancer will experience significant cancer-induced pain, which is disruptive to their quality of life [1]. Unfortunately, persistent pain is often difficult to effectively treat with conventional drugs in bone cancer cases [2]. Although the general characteristic of BCP is similar to inflammatory and/or neuropathic pain in some states, the classical mechanism underlying this chronic pain simply cannot explain the BCP processing, which is triggered by inflammatory, neuropathic and tumorigenic components simultaneously [3-5]. Therefore, it is necessary to investigate the specific molecular and cellular mechanisms by which the cancer cells induce pain sensitization.

Although the origin of BCP is driven initially in the bone microenvironment [6], the neuronal sensitization and glial activation in the spinal cord (SC) level are more far-reaching for contributing to pain hypersensitivity following bone cancer [7]. Accumulating evidence indicates that astrocytes and microglia are activated in the spinal cord dorsal horn (SCDH) after TCI [8] or nerve injury [9]. These activated glial cells are sufficient to trigger the persistent pain hypersensitivity through forming an integrated network of glial-neuronal and/or glial-glial interaction [10]. In particular, the potent inflammatory mediators released from activated glial cells (such as chemokines) have been demonstrated to mediate functional nerve cellular communication, and thereby contribute to persistent pain processing [11,12].

The chemokine CXC motif ligand 12 (CXCL12), formerly named stromal cell-derived factor 1 (SDF-1), is expressed in various kinds of cells in the central nervous system (CNS) and plays an important role in neurogenesis, neuronal migration and neuronal differentiation during the development of the nervous system. The chemokine CXC motif receptor 4 (CXCR4), which belongs to seven-transmembrane-domain G-protein-coupled receptors (GPCRs), is a major type of receptor for CXCL12. CXCL12/CXCR4 chemokine signaling plays a critical role in modulating various nervous system developmental processes and in regulating synaptic plasticity [13]. Dysregulation of this signaling may be an etiological cause for deficient CNS development, oncogenic processes [14] and stroke [15]. Recently, this chemokine signaling has attracted much attention because of its emerging involvement in nociceptive signal regulation. Previous studies have shown that intraplantar inoculation or intrathecal injection of exogenous CXCL12 into naïve rats promote a sustained mechanical allodynia [16,17]. In certain pain-related models, the expression of CXCL12 and/or CXCR4 are significantly increased in the dorsal root ganglion (DRG) or SC, and that blocking CXCR4 can reverse pain-related behaviors [18-20]. Furthermore, CXCL12/CXCR4 signaling has been shown to mediate the induction of TNF-α, NF-κB and IL-6 release from glial cells [21], which could lead to a further nociceptive sensitization. However, it remains largely unknown whether the CXCL12/CXCR4 signaling contributes to pain hypersensitivity in a bone cancer state.

In this study, we set out to investigate whether CXCL12 and CXCR4 are induced in SCDH and participate in BCP by using a rat tibia cancer pain model. We provide the first evidence that the implantation of Walker 256 mammary gland carcinoma cells into a rat tibia produces increased expressions of CXCL12 and CXCR4 in the SC, and that CXCL12/CXCR4 signaling-mediated neuronal sensitization and glial activation are crucial for the development and maintenance of BCP. Furthermore, spinal CXCL12/CXCR4 signaling is now proven to be a potential analgesic target for BCP management.

## Methods

### Animals

Adult female Sprague-Dawley (SD) rats (Experimental Animal Center, Xuzhou Medical College, Xuzhou, China) weighing 180 to 200 g were housed in plastic cages and maintained on a 12:12 hour light/dark circle under conditions of 24°C ± 1°C with food and water available. Before the experiments manipulation, the animals were allowed to habituate in the cages for at least three days. All experimental protocols and animal-handling procedures were performed according to protocols approved by the Animal Care and Use Committee of Xuzhou Medical College, and were consistent with the National Institutes of Health Guide for the Care and Use of Laboratory Animals and the International Association for the Study of Pain’s guidelines for pain research [22]. All efforts were made to minimize the number of animals used and to minimize their suffering.

### Preparation of cells

Walker 256 mammary gland carcinoma cells (Institute for Biomedical Research of Shanghai, Shanghai, China) were derived from SD rats. Ascitic tumor cells (2 × 10^7^ cells/ml, 0.5 ml) were injected into the abdominal cavity of the SD rats weighing 60 to 80 g. After 6 to 7 days, cancerous ascites was extracted in a sterile fashion, and the tumor cells were subsequently washed with sterile normal saline (NS) three times by centrifugation for five minutes at 1000 rpm. The pellet was resuspended with NS and adjusted to an appropriate concentration (1 × 10^5^ cells/μl). The cell suspension was kept on ice until it was injected into rats. For the sham-operated rats, equivolume sterile NS was prepared for injection.

### Model of bone cancer pain

As previously described [23,24], rats were anesthetized with chloral hydrate (400 mg/kg, i.p**.**). The right leg of each rat was shaved and a superficial incision was made in the skin overlying the patella after disinfection with 75% (v/v) ethanol. Then a further incision was cut along the patellar ligament to expose the tibial head with minimal damage. Prepared tumor cells (5 μl) were slowly injected into the right tibia cavity of each rat using a 10 μl microinjection syringe. The syringe was left in place for an additional minute to prevent the tumor cells from leaking out along the injection track. The injection site was closed using bone wax after the syringe was removed to prevent tumor cell overflow. Sham surgery was implemented using a similar procedure by injecting sterile NS (5 μl).

### Drugs and administration

The Jun N-terminal kinases (JNK) inhibitor SP600125 was purchased from Cell Signaling Technology (Danvers, Massachusetts, United States). Anti-CXCL12 neutralizing antibody and anti-IgG antibody (for control) were purchased from Abcam (Cambridge, Massachusetts, United States). Astrocytic inhibitor fluorocitrate and CXCR4 inhibitor AMD3100 were purchased from Sigma Aldrich (St Louis, Missouri, United States). SP600125 were dissolved in dimethyl sulfoxide (DMSO) and diluted in NS (final concentration of DMSO: 1%). Anti-CXCL12 neutralizing antibody and control IgG antibody were dissolved in a sterile artificial cerebrospinal fluid (aCSF) containing 126.6 mM NaCl, 2.5 mM KCl, 2.0 mM MgCl_2_, and 1.3 mM CaCl_2_. Fluorocitrate and AMD3100 were dissolved in saline. The volume for the intrathecal (i.t.) injection was 10 μl. All doses of drugs were based on the results of preliminary experiments. The dose of each drug and time points of treatment were presented in the parts of figure legends.

The method described by Xu JJ *et al.* was used for the intrathecal injection of drugs [25]. Briefly, the rats were anesthetized with isoflurane. The lumbar region was disinfected with 75% (v/v) ethanol after hair shaving, and the intervertebral spaces were widened by placing the animal on a plexiglass tube. Next, a 29-gauge microinjection syringe needle filled with the drug was inserted in the L5-6 interspace. The correct subarachnoid positioning of the tip of the needle was verified by a tail- or paw-flick response immediately after inserting the needle. Then the injection needle was left in place for a further 15 seconds. Motor function was evaluated by the observation of placing or stepping reflexes and righting reflexes at 2 minutes before a nociceptive test. Animals with signs of motor dysfunction were excluded from the experiments.

### Assessment of mechanical allodynia

Mechanical allodynia was determined by measuring the paw withdrawal threshold (PWT) in response to Von Frey hair (Stoelting, Wood Dale, Illinois, United States) stimulation. The protocol was similar to Dixon’s up and down method described by Chaplan SR *et al.*[26]. In briefrats wereindividually placed in a plastic cage with a plexiglass floor and allowed to acclimate for 1 hour before testing. An ascending series of Von Frey hairs (0.4, 0.6, 1.4, 2, 4, 6, 8 and 15 g) were applied to the mid-plantar surface of each hind paw. Each Von Frey hair was held for 5 to 6 seconds with a 5-minute interval between applications. The trial began with the application of the 2 g Von Frey hair. A positive response was defined as brisk withdrawal or paw flinching upon stimulus. Whenever a positive response to a stimulus occurred, the next lower Von Frey hair was applied, and whenever a negative response occurred, the next higher hair was applied. The testing consisted of five more stimuli after the first change in response occurred, and the data were analyzed using the up and down method. The behavioral testing was performed by an investigator blinded to the treatment.

### Assessment of thermal hyperalgesia

Thermal hyperalgesia was determined by measuring the paw withdrawal latency (PWL) in response to radiant heat stimulation. A Plantar Analgesia Meter (IITC Life Science Inc., Victory Blvd Woodland Hills, California, United States) was used to provide a heat source. The protocol was similar to that described by Hargreaves *et al.*[27]. In brief, each rat was placed in a plastic chamber containing a clear glass floor and allowed to acclimatize to the environment for one hour. The radiant heat source was delivered and focused onto the glabrous surface of the paw through the glass floor. An automatic 20 second cutoff was used to prevent tissue damage. Thermal stimulus were delivered three times to each hind paw at 5-minute intervals. The intensity of the heat stimulus was maintained constantly throughout this study.

### Immunohistochemistry

Under deep anesthesia, rats were intracardially perfused with phosphate-buffered saline (PBS) followed by 4% paraformaldehyde (PFA). Then the L4-5 spinal cord segment was dissected and post-fixed in 4% PFA for 3 hours, and subsequently allowed to equilibrate in 30% sucrose in a phosphate buffer (PB) overnight at 4°C. The embedded blocks were sectioned 30 μm thick in a cryostat and stored in PBS for immunofluorescence. The sections were first blocked with 5% donkey serum and 0.3% Triton X-100 for 1hour at room temperature, then incubated overnight at 4°C with the following primary antibodies: rabbit anti-CXCL12 polyclonal antibody (1:100, sc-28876, Santa Cruz Biotechnology, Santa Cruz, California, United States), rabbit anti-CXCR4 polyclonal antibody (1:200, ab2074, Abcam, Cambridge, Massachusetts, United States), mouse anti-NeuN monoclonal antibody (1:400, Alexa Fluor 488 Conjugate, MAB377X, Millipore, Billerica, Massachusetts, United States), mouse anti-GFAP monoclonal antibody (1:400, Alexa Fluor 488 Conjugate, #3655, Cell Signaling Technology, Beverly, Massachusetts, United States), goat anti-IBA1 polyclonal antibody (1:400, ab5076, Abcam, Cambridge, Massachusetts, United States ), rabbit anti-c-Fos polyclonal antibody (1:800, ab7963, Abcam, Cambridge, Massachusetts, United States ). The sections were then washed 3 times with PBS and incubated with the specific secondary antibodies raised in donkey serum(conjugated to Alexa Fluor 488 or 594, Invitrogen, Carlsbad, California, United States) overnight at 4°C. For double staining, sections were incubated again in the same blocking solution and the staining process was performed once again. After immunostaining procedures, the sections were examined using a laser scanning confocal microscopy (FV1000, Olympus, Tokyo, Japan).

According to the previous description [28,29], c-Fos immunoreactive neurons were counted in a blind fashion. The number of c-Fos like immunoreactive neurons in the dorsal horn of the spinal cord (laminae I-VII) was determined by averaging the counts made in 30 spinal cord sections for each group (n = 5). To obtain quantitative measurements of GFAP and IBA1 immunofluorescence in the dorsal horn, 15 to 20 fields covering the entire dorsal horn in each group (n = 5) were evaluated and photographed at the same exposure time to generate the raw data. The average green fluorescence intensity of each pixel was normalized to the background intensity in the same image.

### Western blot analysis

The L4-5 spinal cords and DRGs were quickly extracted from deeply anesthetized rats, then the tissue samples were dissected and stored in liquid nitrogen. The tissues were homogenized in a lysis buffer (Bio-Rad Laboratories, Hercules, California, United States) containing a cocktail of protease inhibitor and phosphatase inhibitors (Sigma Aldrich, St Louis, United States). Then homogenates were centrifuged at 12000 g for 15 minutes at 4°C. The supernatants of the homogenates were collected and the protein concentration of the supernatants was estimated using the method of bicinchoninic acid assay. The total protein content among samples was equalized. The samples were dissociated by heating at 100°C for five minutes in a loading buffer (2% sodium dodecyl sulfate, 100 mM dithiothreitol, 10% glycerol, and 0.02% bromophenol blue). Then equivalent amounts of protein (80 μg) were separated using 10% sodium dodecyl sulfate-polyacrylamide gel electrophoresis (SDS-PAGE) and transferred onto a polyvinylidene fluoride membrane (PVDF, Millipore, Billerica, Massachusetts, United States). The membranes were placed in a blocking solution, which contained Tris-buffered saline with 0.02% Tween (TBST) and 5% non-fat dry milk, for two hours at room temperature, and then incubated overnight at 4°C with rabbit anti-CXCL12 polyclonal antibody (1:400, sc-28876, Santa Cruz Biotechnology, California, United States), rabbit anti-CXCR4 polyclonal antibody (1:1000, ab2074, Abcam, Cambridge, Massachusetts, United States) and glyceraldehyde-3-phosphate dehydrogenase (GAPDH, 1:50000, G9545, Sigma, St Louis, Missouri, United States) , respectively.

Data were analyzed with Quantity One Analysis Software (Version 4.6.5, Bio-Rad Laboratories, Hercules, California, United States). Each of the groups was first standardized with the control GAPDH. The mean value of the sham group (n = 5) was set as 1, which was used for normalization for each of the 5 sham groups. For each of the molecules detected (n = 5), the sham group (set as 1) was used for normalization of the protein expression in the TCI groups at every data points after different treatments. The results were showed in the figures as fold changes.

### Statistical analyses

GraphPad Prism 5 (GraphPad Software, La Jolla, California, United States) was used to conduct all statistical analyses. Alterations of detected protein expression and changes of behavioral responses to mechanical and thermal stimuli over time among groups were tested using one-way analysis of variance (ANOVA) with repeated measures, followed by the Dunnett multiple comparison test, and two-way ANOVA with repeated measures, followed by Bonferroni *post hoc* test, respectively. All data are presented as means ± SEM. Statistical results are considered significant if *P* < 0.05.

## Results

### Spinal CXCL12 is upregulated in astrocytes after TCI

**Figure 1:**
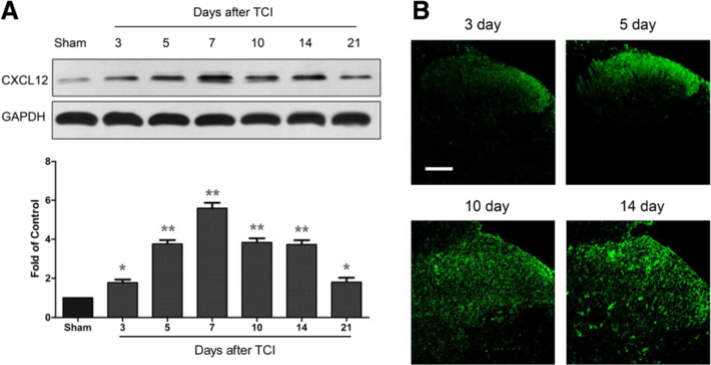
**Expression and distribution of CXCL12 protein in the spinal cord after TCI in rats. (A)** Western blot analysis shows time course of CXCL12 expression in sham and TCI rats. Five spinal cord segments were included in each group. Representative bands are shown on the top; data summary is shown on the bottom. *P < 0.05, **P < 0.01 versus sham control. **(B)** Immunofluorescence shows expression of CXCL12 protein (green) in rat spinal cord at different time points. Tissues were collected at day 3, 5, 10, 14 after TCI. Original magnification: 200× for all the confocal images showing the dorsal horn ipsilateral to TCI (bar = 100 μm).

Bone cancer-induced chemokine changes in the SC are critical for BCP generation [30-33]. Here we examined whether CXCL12/CXCR4 chemokine signaling could be functional upregulated in BCP state. We first detected the expression and distribution of CXCL12 in the SC after TCI. Our western blot analysis showed that TCI induces a rapid-onset and long-lasting increased expression of CXCL12 protein from day 3 to day 21 after TCI. The increase evidently began at day 3, peaked at day 7 to 10, and remained at high level until day 21, the last test day (Figure 1A). However, CXCL12 protein was at a low level in the SC of sham-operated rats. In addition, the immunoreactivity of CXCL12 at the start was distributed predominantly in the superficial layers (laminae I-II) at day 3 and 5 after TCI. However, CXCL12 was not restricted to the superficial dorsal horn (DH) for the whole time. A widespread induction of CXCL12 was observed in SCDH at day 10 and 14 after TCI (Figure 1B).

**Figure 2:**
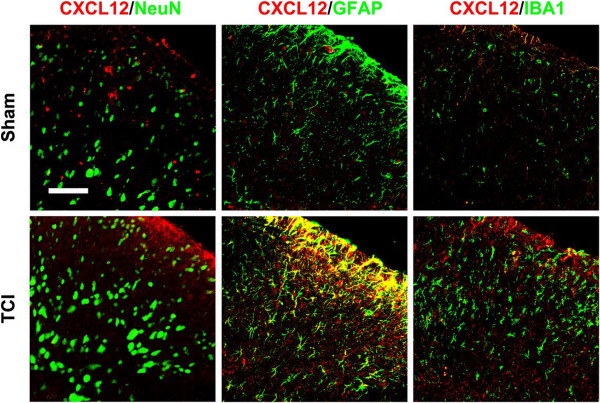
**Cellular localization of CXCL12 expression in spinal cord dorsal horn of sham and TCI rats.** Double staining shows that CXCL12 (red) is colocalized with GFAP (green, middle), a marker for astrocytes, but not with NeuN (green, left), a marker for neurons or IBA1 (green, right), a marker for microglia. Tissues were collected 10 days after TCI. Two single-stained images were merged. Original magnification: 400× for all the confocal images showing the dorsal horn ipsilateral to TCI (bar = 50 μm).

To investigate the cell distribution of CXCL12 in DH following TCI treatment, we performed double immunostaining of CXCL12 with three major spinal nerve cell-specific markers: NeuN (for neurons), GFAP (for astrocytes), and IBA1 (for microglia). Confocal images showed that GFAP and IBA1 were expressed at a low immunoreactivity in SCDH of sham-operated rats, but were increased at day 10 after TCI, indicating the activation of astrocytes and microglia was caused by TCI. Furthermore, the double immunostaining of CXCL12 was extensively colocalized with GFAP, but hardly colocalized with NeuN or IBA1, suggesting that CXCL12 is induced by astrocytes, but not neurons or microglia in BCP rats (Figure 2). These results demonstrate that spinal CXCL12 is upregulated after TCI, and predominately expressed in activated astrocytes.

### CXCL12 is also increased in DRG after TCI

**Figure 3:**
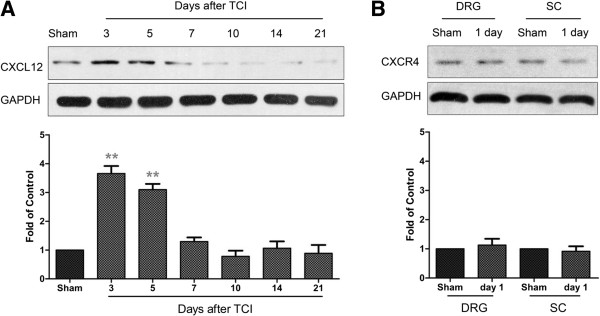
**Expression of CXCL12 protein in DRG after TCI in rats. (A)** Western blot analysis shows time course of DRG CXCL12 expression in sham and TCI rats. **(B)** Western blot analysis shows DRG or SC CXCL12 expression in sham and TCI rats at day 1. Five samples were included in each group. Representative bands are shown on the top; data summary is shown on the bottom. **P < 0.01 versus sham control.

To investigate whether, besides spinal astrocytes, DRG would be another source for the spinal CXCL12 production after TCI, we further tested the CXCL12 expression in DRG level. Our western blot analysis showed that TCI induces a rapid-onset but short-duration upregulation of DRG CXCL12 expression after TCI. The CXCL12 evidently increased on day 3 and day 5, then rapidly decreased to a normal level from day 7 to day 21 (Figure 3A). This data suggested that the increased CXCL12 in the spinal cord at the early phase may be coming from DRG through axonal transportation. However, neither spinal nor DRG CXCL12 expression was statistically increased at day 1 after TCI (Figure 3B).

### TCI induces CXCL12 upregulation in spinal astrocytes via JNK pathway

**Figure 4:**
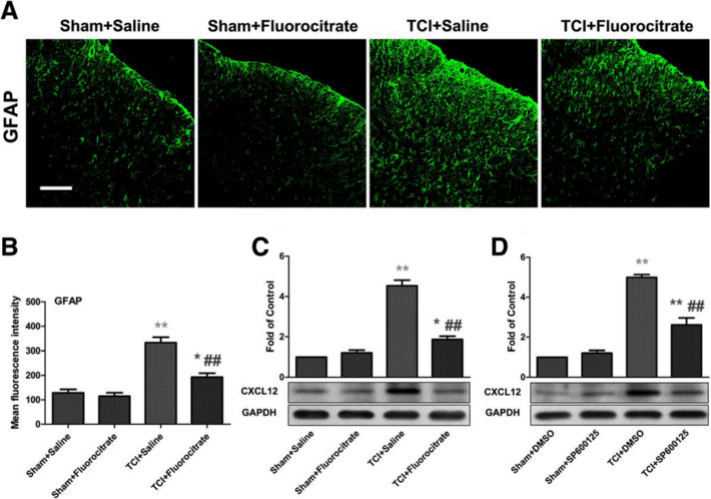
**Intrathecal administration of fluorocitrate or SP600125 reverses TCI-induced upregulation of CXCL12 protein in the spinal cord. (A)** Confocal images show inhibitory effect of fluorocitrate on induction of astrocytic marker GFAP (green) after TCI. Original magnification: ×200. **(B)** Mean green immunofluorescence intensity of GFAP of **A** among sham, TCI and TCI + Fluorocitrate group. For control experiments, saline was used. Fluorocitrate (1 nmol/10 μl, i.t.) or saline (10 μl, i.t.) was given once a day on postoperative days 8, 9 and 10, respectively. Tissues were collected 4 hours after the last spinal injection (n = 5 in each group). *P < 0.05, **P < 0.01 versus sham + saline. ##*P* < 0.01 versus TCI + saline. **(C)** Western blot and data summary show inhibitory effect of fluorocitrate on TCI-induced increased expression of CXCL12 protein (n = 4 in each group). **(D)** Western blot and data summary show inhibitory effect of SP600125 on TCI-induced increased expression of CXCL12 protein (n = 4 in each group). SP600125 (20 nmol/10 μl, i.t.) or DMSO (for control, 10 μl, i.t.) was given once a day on postoperative days 8, 9 and 10, respectively. Tissues were collected 6 hours after the last spinal injection. **P < 0.01 versus sham + DMSO. ##*P* < 0.01 versus TCI + DMSO.

To examine whether the production of CXCL12 is required for the activation of astrocytes, we intrathecally injected an astrocyte metabolic inhibitor fluorocitrate [34] (1 nmol/10 μl) into the bone cancer rats once daily for three consecutive days (from day 8 to 10 after TCI), then detected the GFAP immunoreactivity and CXCL12 protein level after the last injection. Compared with sham-operated rats, the immunofluorescence intensity of GFAP and expression level of CXCL12 were increased at day 10 in TCI-treated rats. After repeated fluorocitrate administration, however, the TCI-induced GFAP immunofluorescence expression was abolished (Figure 4A and B). Further more, the TCI-induced CXCL12 protein upregulation was also inhibited when associated with GFAP decrease (Figure 4C). In contrast, saline did not affect GFAP immunofluorescence intensity or CXCL12 protein expression in BCP rats.

The JNK is known to activate in spinal astrocytes after nerve injury [35] or bone cancer [36], and JNK activation appears to regulate the production of certain chemokines in astrocytes [37,38]. To further define whether JNK is the upstream of CXCL12 after TCI, we blocked the JNK pathway with the selective JNK inhibitor SP600125 [37] (20 nmol/10 μl, i.t. once daily from day 8 to 10 after TCI) and then detected CXCL12 protein level after the last injection. Compared with the control DMSO, SP600125 reversed TCI-induced CXCL12 increase after three consecutive days of i.t. administration (Figure 4D). Collectively, these findings suggest that JNK pathway mediates TCI-induced production of spinal CXCL12 in activated astrocytes.

### CXCL12 neutralizing antibody attenuates mechanical allodynia and thermal hyperalgesia caused by TCI

**Figure 5:**
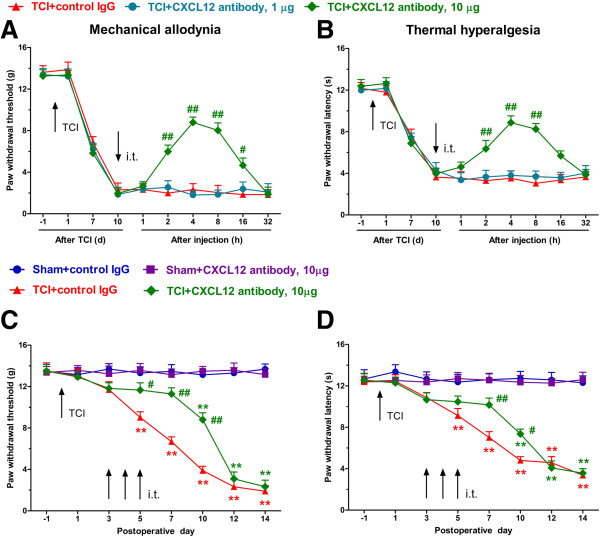
**Effects of CXCL12 neutralizing antibody in spinal cord on mechanical and thermal hypersensitivity induced by TCI. (A, B)** Intrathecal injection of CXCL12 neutralizing antibody (10 μg, but not 1 μg) partially and transiently reverses TCI-induced mechanical allodynia **(A)** and thermal hyperalgesia **(B)** in the feet ipsilateral to TCI. Pain behaviors were measured on postoperative day 10. #*P* < 0.05, ##*P* < 0.01 versus TCI + control IgG. **(C, D)** Intraspinal injection of CXCL12 neutralizing antibody (10 μg/10 μl, once a day for three consecutive days on days 3, 4, and 5 after TCI) delays TCI-induced induction of mechanical allodynia **(C)** and heat hyperalgesia **(D)**. **P < 0.01 versus sham + control IgG. #*P* < 0.05, ##*P* < 0.01 versus TCI + control IgG. TCI was performed on day 0. The dose of control IgG was 10 μg/10 μl. Each administration is indicated by an arrow on the corresponding time point. Ten rats were included in each group.

To elucidate whether suppressing spinal CXCL12 could inhibit pain-related behaviors following TCI, we intrathecally injected a CXCL12 neutralizing antibody [39] into sham and TCI-operated rats. We first tested the analgesic effect of acute CXCL12 inhibition by a single i.t. injection of CXCL12 neutralizing antibody at day 10 after TCI. At this time point, the pain-related behaviors are well-established and the upregulation of CXCL12 remains at a high level. The TCI significantly decreased PWT and PWL in the ipsilateral side at day 10. Compared with control IgG (10 μg/10 μl) treatment, BCP rats treated with CXCL12 neutralizing antibody, at the dose of 10 μg (but not 1 μg), showed a transient recovery in the decreased levels of mechanical threshold and thermal latency at 2, 4, 8, 16 hours after antibody administration (Figure 5A and B). These results indicate that CXCL12 may be involved in the maintenance of BCP.

Considering the upregulated expression of spinal CXCL12 began at day 3, about two days prior to the detectable onset of cancer pain-related behaviors, we hypothesized that TCI-induced rapid-onset increase of endogenous CXCL12 may contribute to the induction of BCP. In support of our hypothesis, we further investigated the analgesic effect of CXCL12 neutralizing antibody on pain behavior induction. The behavioral data showed that repeated treatment of CXCL12 neutralizing antibody (10 μg i.t., once a day from day 3 to 5 after TCI) for three continuous days significantly delayed the onset of mechanical allodynia and thermal hyperalgesia for almost five days (Figure 5C and D). To test the effect of control IgG and CXCL12 neutralizing antibody on basal nociceptive condition, we i.t. injected IgG or CXCL12 neutralizing antibody (10 μg/10 μl) in sham rats, and then tested the PWT and PWL. The behavioral results showed that neither control IgG nor CXCL12 neutralizing antibody affect the PWT or PWL from day 3 to 14 after TCI. Taken together, these behavioral results provide strong evidence that CXCL12 in the spinal cord is critical for TCI-induced pain sensitization.

### Expression and distribution of CXCR4 in spinal cord after TCI

**Figure 6:**
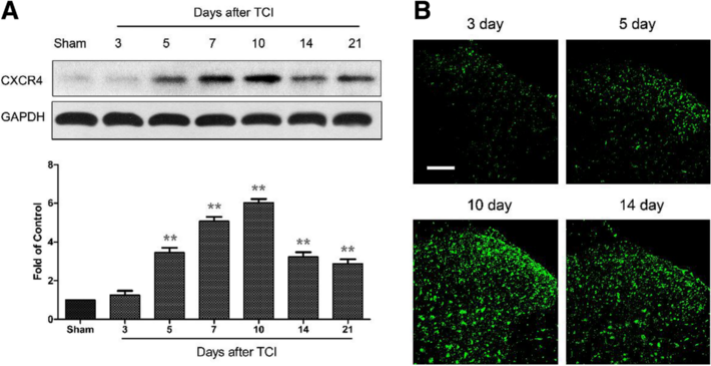
**Expression and distribution of CXCR4 protein in spinal cord after TCI in rats. (A)** Western blot analysis shows time course of CXCR4 expression in sham and TCI rats. Five spinal cord segments were included in each group. Representative bands are shown on the top; data summary is shown on the bottom. **P < 0.01 indicate significant differences compared with group of sham control. **(B)** Immunofluorescence shows expression of CXCR4 protein (green) in rat spinal cord at different time points. Tissues were collected at day 3, 5, 10 and 14 after TCI. Original magnification: 200× for all the confocal images showing the dorsal horn ipsilateral to TCI (bar = 100 μm).

CXCR4 is a major type of receptor for CXCL12. We further investigated whether the spinal CXCR4 could be triggered after CXCL12 upregulation in BCP condition. Compared with the rapid-onset of CXCL12, the increased expression of its receptor CXCR4 delayed by two days, then peaked at day 7 to day 10, and remained at a high level until day 21. Whereas, the expression of CXCR4 protein was also at a low level in sham rats (Figure 6A). Additionally, our immunofluorescence result showed that CXCR4 predominantly distributed in the superficial layers at day 3 and 5 after TCI. However, this is followed by a widespread induction of CXCR4 both in the surface and deeper layers at day 10 and 14 after TCI (Figure 6B).

**Figure 7:**
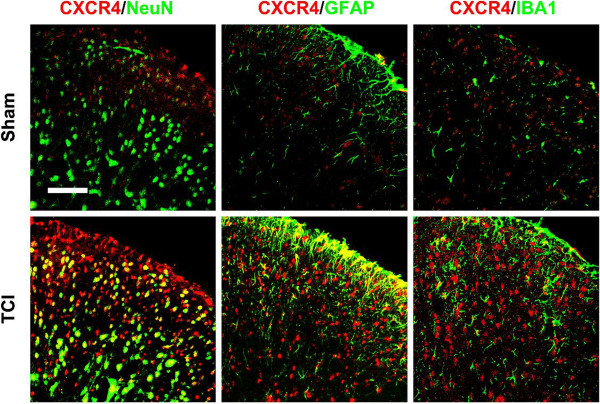
**Cellular localization of CXCR4 expression in spinal cord dorsal horn of sham and TCI rats.** Double staining shows that CXCR4 protein (red) is co-expressed with neuronal marker NeuN (green, left), astrocytic marker GFAP (green, middle), and microglial marker IBA1 (green, right). Tissues were collected 10 days after TCI. Original magnification: 400× for all the confocal images showing the dorsal horn ipsilateral to TCI (bar = 50 μm).

Given that the localization of CXCR4 determines the functional targets of CXCL12/CXCR4 signaling, we then examined the cellular distribution of CXCR4 with NeuN, GFAP and IBA1 in SCDH of TCI or sham rats. Confocal images showed that after TCI treatment immunoreactivity of CXCR4 was predominantly colocalized with neurons and astrocytes, and a small amount with microglia. However, the immunoreactivity of CXCR4, GFAP and IBA1 in the sham rats was quite weak (Figure 7).

### Blocking CXCR4 suppresses TCI-induced activation of neurons and glial cells

**Figure 8:**
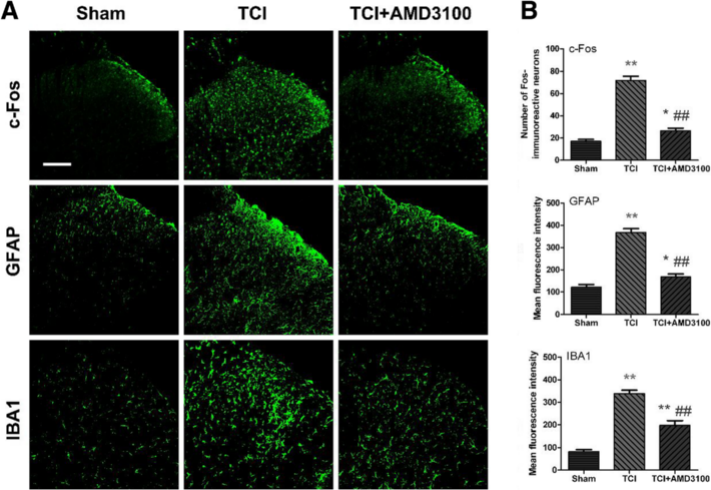
**AMD3100 suppresses TCI-induced upregulation of c-Fos, GFAP and IBA1 in spinal cord dorsal horn. (A)** Confocal images shows activation of neurons (c-Fos), astrocytes (GFAP) and microglia (IBA1) are suppressed by intraspinal injection of AMD3100. Original magnification: 200× for all the confocal images showing the dorsal horn ipsilateral to TCI (bar = 100 μm). **(B)** Data summary of **A**. AMD3100 (5 μg/10 μl, i.t.) was given once a day on postoperative days 12, 13 and 14, respectively. Tissues were collected 6 hours after the last injection (30 spinal cord sections in each group). *P < 0.05, **P < 0.01 versus sham; ##*P* < 0.01 versus TCI.

TCI causes neurochemical alterations such as c-Fos, GFAP and IBA1 in the ipsilateral DH, in addition to behavioral changes of bone cancer [40]. To reveal the cellular consequences after CXCR4 upregulation, we continued to investigate whether CXCR4 would be involved in the activation of neurons (c-Fos), astrocytes (GFAP) and microglia (IBA1) caused by TCI. Our immunofluorescence images showed that TCI-induced induction of c-Fos and activation of GFAP and IBA1 were all greatly suppressed by AMD3100 (5 μg/10 μl, i.t., once a day from day 12 to 14 after TCI) for three consecutive days. The results indicate that spinal CXCR4 may directly mediate the sensitization of neurons and activation of astrocytes and microglia in the BCP condition (Figure 8).

### AMD3100 prevents and alleviates bone cancer pain in the early- and late-phase

**Figure 9:**
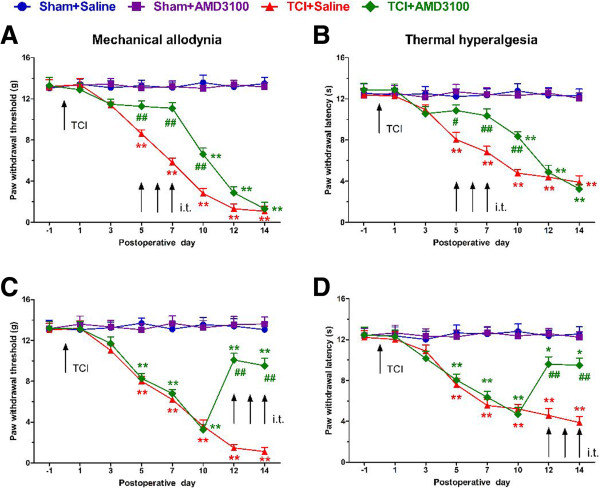
**Intrathecal administration of AMD3100 prevents and attenuates bone cancer pain after TCI in rats. (A, B)** Intrathecal administration of AMD3100 (5 μg/10 μl, i.t., once a day) in the early phase (postoperative days 5, 6 and 7) delayed the onset of mechanical allodynia **(A)** and thermal hyperalgesia **(B)** in TCI rats. **(C, D)** In the later phase (postoperative days 12, 13 and 14), AMD3100 (5 μg/10 μl, i.t., once a day) suppressed the ongoing mechanical allodynia **(C)** and thermal hyperalgesia **(D)**. TCI was done on day 0. Each administration is indicated by an arrow on the corresponding time point. Ten rats were included in each group. *P < 0.05, **P < 0.01 versus sham + saline. #*P* < 0.05, ##*P* < 0.01 versus TCI + saline.

Previous reports have shown that systematic administration of AMD3100 attenuates the pain hypersensitivity in some rat models of neuropathy [18-20]. Here, we first reported the analgesic effects of AMD3100 on bone cancer-induced pain in TCI-operated rats. As the capability of AMD3100 on crossing the blood–brain barrier is not clear, we employed the method of intrathecal injection to observe the direct effects of this drug at the spinal level. The drug dosage was determined by preliminary experiments. Repetitive i.t. administration of AMD3100 (5 μg/15 μl, once a day for three days) significantly delayed the initiation of mechanical allodynia and thermal hyperalgesia for three days at the early phase (day 5, 6 and 7 after TCI) of bone cancer (Figure 9A and B). Furthermore, established mechanical allodynia and thermal hyperalgesia were also significantly reversed by repetitive i.t. administration of the same dose of AMD3100 at the late phase (day 12, 13 and 14 after TCI) of bone cancer (Figure 9C and D). While normal pain sensation in the sham rats was not altered by AMD3100 or saline, these results indicate that CXCL12/CXCR4 signaling indeed plays an important role in the production and persistence of BCP.

## Discussion

Neuronal sensitization associated with glial activation in SC has been shown to be important in the development and maintenance of pain hypersensitivity in bone cancer state. There is growing evidence that chemokines (such as CCL2, CCL5, CXCL1 and CX3CL1) are involved in neuronal sensitization and/or glial activation in SC [11,38,41], and some of them contribute to BCP processing [30,42]. In this study, we characterized the expression, distribution and function of chemokine CXCL12 and its receptor CXCR4 in TCI model of BCP. The principal findings of our study are: (1) TCI induced a rapid-onset and long-lasting upregulation of CXCL12 in SC, which is dependent on astrocytes activation and JNK pathway. (2) A single i.t. injection of CXCL12 neutralizing antibody transiently reversed established-pain hypersensitivity induced by TCI. Additionally, repetitive i.t. injection of CXCL12 neutralizing antibody persistently delayed the induction of TCI-induced pain hypersensitivity. (3) TCI also induced upregulation of CXCR4 protein in SC, which is expressed in neurons, astrocytes and microglia. (4) Repeated i.t. administration of AMD3100, a selective CXCR4 inhibitor, suppressed TCI-induced activation of neurons, astrocytes and microglia in SCDH. (5) AMD3100 prevented and alleviated pain hypersensitivity following TCI in early and later phase of bone cancer. These findings suggest that spinal CXCL12/CXCR4 signaling plays an important role in the development and maintenance of BCP.

To date, a growing body of evidence in rat experiments shows that CXCL12 is expressed at key locations of the pain transmission pathway in pathological pain states. In the DRG level, nucleoside reverse transcriptase inhibitors and prolonged morphine administration both resulted in increased mechanical allodynia and CXCL12 mRNA upregulation [18,20]. In addition, CXCL12 mRNA in the satellite glial cells was induced for 14 days following chronic constriction injury [19]. In the SC level, CXCL12 immunoreactivity was also induced after spinal cord injury, and co-expressed with pain-related peptides: substance P and calcitonin gene-related peptide (CGRP) [43]. By contrast, our results from western blot detection showed that TCI induced a rapid (3 days) and persistent (21 days) increase of CXCL12 protein in the SC. Furthermore, it was previously reported in an *in vitro* study that CXCL12 is expressed intensely in astrocytes and weakly in neurons, but not in microglia [44]. In our *in vivo* study, immunofluorescence double staining detection also showed that after TCI CXCL12 was increased and predominantly expressed in astrocytes, but only occasionally in neurons or microglia.

Astrocytes activation, referred to GFAP upregulation and hypertrophy, was detected in various pathological pain conditions, and was generally considered to be responsible for enhancing persistent pain states [34]. In the CNS, astrocytes have been identified as sources of algogenic substance, because accumulating evidence indicates that activated astrocytes can release pro-inflammatory cytokines (such as IL-1β and TNF-α) and chemokines (such as CCL2 and CXCL1) in the SC to enhance and prolong pain processing [38,45-47]. We showed that fluorocitrate, which disrupts astrocytes function, exerted a profound blockade of CXCL12 induction in bone cancer states. These data indicate that chemokine CXCL12 was also released from activated astrocytes. Furthermore, JNK, one of the members of MAPK, is highly expressed in activated astrocytes and regulates the production and release of various chemokines (such as CCL2 and CXCL1) in neuropathic pain conditions. In this study, we further found that JNK inhibitor SP600125 reduced TCI-induced CXCL12 production, indicating that JNK is a critical upstream trigger for CXCL12 upregulation in astrocytes after TCI.

Interestingly, our results showed that CXCL12 induction started detectable from day 3 after TCI. However, GFAP was not activated until day 7 [48], suggesting that CXCL12 may be also produced and released from other cells in the early phase of bone cancer. Indeed, in a basal condition CXCL12 was predominantly distributed in both peptidergic CGRP and the non-peptidergic isolectin B4 (IB4) nociceptive neurons of DRG, and also found in CGRP nociceptive terminals within DH, demonstrating that CXCL12 was broadly expressed in the nociceptive pathways. Therefore, we thought it necessary to detect the CXCL12 expression in DRG level after TCI, in order to explain the increase of CXCL12 in SC before astrocytes activation. Our western blot analysis showed that TCI induces a rapid-onset but short-duration upregulation of CXCL12 expression in the DRG level at early phase. In addition, our confocal images also showed that, at the very start after TCI, spinal CXCL12 was distributed predominantly in the superficial layers, which is the important region for processing nociceptive information from DRG to SC. As time goes on, however, a widespread induction of CXCL12 was observed in the whole SCDH after astrocytes were activated. We thus hypothesized that, in bone cancer state (especially in the early phase), CXCL12 is produced in DRG nociceptive neurons that innervate the tumor-bearing limb, and in turn axonally transported to the surface layers of SCDH where it may be released locally. While, in the later phase, CXCL12 is mainly produced and released by spinal astrocytes, considering that, at this time, DRG CXCL12 has been decreased to a normal level and spinal astrocytes have been activated and distributed widely.

It is becoming clear that CXCL12 is produced from activated astrocytes via JNK pathway on bone cancer status. However, whether CXCL12 contributes to bone cancer pain depends on behavioral examination. In support of this notion, we demonstrated for the first time, to our knowledge, that the CXCL12 neutralizing antibody can alleviate established cancer pain hypersensitivity by i.t. administration, despite that the effect of anti-hyperalgesia and anti-allodynia is transient. Certainly, our western blot result showed that CXCL12 upregulation started from day 3 after TCI, while the cancer pain hypersensitivity was undetectable until day 5, suggesting that spinal CXCL12 may be a trigger mediator for development of BCP. As expected, we further found that prolonged treatment of CXCL12 neutralizing antibody in the early phase of bone cancer markedly delayed the onset of BCP behavior. Thus, our data supports a potential role of CXCL12 in the development of BCP processing.

All chemokines exert their biological functions via activating their surface receptors which are on different types of cells. Annabelle *et al.* have reported that exogenous CXCL12 directly induced mechanical hypersensitivity in naïve rats through the stimulation of CXCR4 in the SC [16]. Consistently, CXCR4 receptors have been observed both in DRG and SC, and implicated in CXCL12-mediated pain regulation after neuropathy. Remarkably, we found that CXCR4 was significantly and persistently upregulated accompanying CXCL12 production in SC after TCI. It is becoming clear that, through releasing several chemokines, the activated glial cells not only enhance neuronal sensitization, but also further facilitate glial activation, and finally enhance pathological pain processing. Such positive feedback loops, which are consist of glial-neuronal and glial-glial communication, depend on both perseverant release of chemokines from glial cells and persistent activation of chemokine receptors on neurons and glial cells [49]. In the present study, after confirming the origin of CXCL12, we further observed that the increased CXCR4 was widely distributed around the neurons, astrocytes and microglia as well, which is in parallel with previous studies in pathological conditions. This find indicated that CXCL12/CXCR4 signaling may be making major contributions to nociceptive signal processing by mediating glial-neuronal and glial-glial communication on bone cancer state. Given the spinal blockade of CXCR4 inhibits TCI-induced induction of c-Fos and activation of GFAP and IBA1 in SCDH, we further confirmed that CXCL12/CXCR4 signaling contributes to a positive feedback loop of glial-neuronal/glial communication through directly activating neurons and glial cells after TCI, which are essential to BCP regulation. In addition, CXCR4 receptors activate various signaling pathways, such as the mitogen-activated protein kinase (MAPK) pathway, phospholipase C (PLC) pathway, and phosphatidyl inositol-3 kinase (PI3K) pathway, which leads to varied functional outcomes, including adhesion, polarization, and chemotaxis [50,51]. These intracellular signaling pathways are also involved in nociceptive regulation [52-54]. However, whether CXCL12/CXCR4 signaling mediates BCP processing through MAPK and/or PLC and/or PI3K pathway in spinal glia needs to be investigated in the future.

As a specific antagonist of chemokine receptor CXCR4, AMD3100 has been approved in human clinical trials for hematopoietic stem cell mobilization to the peripheral blood in patients with non-Hodgkin’s lymphoma and multiple myeloma [55,56]. It has also been demonstrated in animal models that AMD3100 as a novel therapeutic concept has been applied, particularly for the treatment of tumor progression including angiogenesis, metastasis, survival and bone destruction [57-59]. Our behavioral results further demonstrated that spinal administration of AMD3100 attenuated TCI-induced pain hypersensitivity at the early induction phase and late maintenance phase, suggesting the analgesic effect of AMD3100 on BCP. If AMD3100 can block tumor growth, tumor-induced bone destruction and BCP without substantial side effects, treatment with AMD3100 may considerably enhance the quality of life for patients with primary or metastatic bone cancer.

## Conclusions

In conclusion, our present study suggests that implantation of Walker 256 mammary gland carcinoma cells into the tibia of rats produces a prominent expression of CXCL12/CXCR4 in the spinal cord, which may underlie the activation of these spinal neurons and glial cells as well as hyperalgesic behaviors in bone cancer rats. Thus, the blockade of this chemokine signaling in the spinal cord may play a vital role in bone cancer pain management.

## Abbreviations

aCSF: Artificial cerebrospinal fluid; ANOVA: Analysis of variance; BCP: Bone cancer pain; CGRP: Calcitonin gene-related peptide; CNS: Central nervous system; CXCL12: Chemokine CXC motif ligand 12; CXCR4: Chemokine CXC motif receptor 4; DMSO: Dimethyl sulfoxide; DRG: Dorsal root ganglion; GAPDH: Glyceraldehyde-3-phosphate dehydrogenase; GFAP: Glial fibrillary acidic protein; GPCR: G-protein-coupled receptor; IB4: Isolectin B4; IBA1: Ionized calcium binding adapter molecule-1; IL: Interleukin; i.t.: Intrathecal; i.p.: Intraperitoneal; JNK: Jun N-terminal kinases; MAPK: Mitogen-activated protein kinase; NeuN: Neuronal nucle; NS: Normal saline; PB: Phosphate buffe; PBS: Phosphate-buffered saline; PLC: Phospholipase C; PI3K: Phosphatidyl inositol-3 kinase; PVDF: Polyvinylidene fluoride membrane; PWL: Paw withdrawal latency; PWT: Paw withdrawal threshold; SD: Sprague-Dawley; SDF-1: Stromal cell-derived factor 1; SEM: Standard error of the mean; TCI: Tumor cell implantation; TNF: Tumor necrosis factor.

## Competing interests

The authors declare that they have no competing interests.

## Authors’ contributions

WS conceived and designed the study. XMH performed the animal surgery, behavioral testing and data analysis. YNL carried out the immunohistochemistry and western blot experiments. YH coordinated and supervised the experiments. LPC, CCW and CS participated in part of the behavioral testing and immunohistochemistry experiments. All authors read and approved the final manuscript.
